# Surveillance for Colonization, Transmission, and Infection With Methicillin-Susceptible *Staphylococcus aureus* in a Neonatal Intensive Care Unit

**DOI:** 10.1001/jamanetworkopen.2021.24938

**Published:** 2021-09-13

**Authors:** Dennis Nurjadi, Vanessa M. Eichel, Patrik Tabatabai, Sabrina Klein, Katharina Last, Nico T. Mutters, Johannes Pöschl, Philipp Zanger, Klaus Heeg, Sébastien Boutin

**Affiliations:** 1 Department of Infectious Diseases, Medical Microbiology and Hygiene, Heidelberg University Hospital, Heidelberg, Germany; 2 Department of Neonatology, Heidelberg University Children’s Hospital, Heidelberg, Germany; 3 Centre for Infectious Diseases, Institute of Medical Microbiology and Hygiene, Saarland University, Homburg, Germany; 4 Institute for Hygiene and Public Health, Bonn University Hospital, Bonn, Germany; 5 Heidelberg Institute of Global Health, Heidelberg University Hospital, Heidelberg, Germany

## Abstract

**Importance:**

*Staphylococcus aureus* is one of the leading causes of infections in neonatal intensive care units (NICUs). Most studies in this patient group focus on methicillin-resistant *S aureus* or the outbreak setting, whereas data for methicillin-susceptible *S aureus* are limited.

**Objectives:**

To identify risk factors for *S aureus* colonization and infections in hospitalized newborns and to investigate *S aureus* transmission and its dynamics in a nonoutbreak setting.

**Design, Setting, and Participants:**

This monocentric cohort study in a tertiary NICU in Heidelberg, Germany, enrolled all hospitalized neonates (n = 590) with at least 1 nasal screening swab positive for *S aureus*. Data were collected from January 1, 2018, to December 31, 2019.

**Exposures:**

Weekly screening for *S aureus* colonization was performed for all newborns until discharge.

**Main Outcomes and Measures:**

The primary end point was any *S aureus* infection until hospital discharge. Transmission of *S aureus* and performance of routine typing to detect transmissions were defined as the secondary outcomes of the study.

**Results:**

In total, 590 newborns were enrolled (276 [46.8%] female and 314 [53.2%] male; 220 [37.3%] with birthweight <1500 g; 477 [80.8%] preterm; 449 [76.1%] singletons; 419 [71.5%] delivered via cesarean section). The median length of stay was 26 (range, 10-62) days. Overall, 135 infants (22.9%) were colonized by *S aureus* at some time during their hospital stay. The median time to first detection was 17 (interquartile range, 11-37) days. The overall incidence of *S aureus* infection was 1.7% (10 of 590). Low birth weight (<1500 g [odds ratio, 9.3; 95% CI, 5.9-14.6; *P* < .001]) and longer hospital stay (odds ratio, 2.3; 95% CI, 1.9-2.7; *P* < .001) were associated with colonization. Nasal carriage was significantly associated with *S aureus* infection (odds ratio, 8.2; 95% CI, 2.1-32.3; *P* = .002). A total of 123 of 135 colonization isolates were sequenced. All recoverable infection isolates (4 of 7) of newborns with colonization were genetically identical to the colonizing isolate. Whole-genome sequencing indicated 23 potential transmission clusters.

**Conclusions and Relevance:**

The findings of this cohort study suggest that nasal colonization is a relevant risk factor for *S aureus* infection in a nonoutbreak NICU setting. In colonized newborns, infection and colonization isolates were genetically identical, suggesting that eradication of colonization may be a useful measure to prevent infection. Further investigations are necessary to validate and assess the generalizability of our findings.

## Key Points

**Question:**

What are the risk factors for the acquisition of methicillin-susceptible *Staphylococcus aureus* (MSSA) colonization and infections in a neonatal intensive care unit?

**Findings:**

In this cohort study of 590 newborns, the acquisition of *S aureus* colonization and infection was monitored until hospital discharge. Colonization with MSSA was associated with lower birth weight, longer hospitalization, and higher odds of acquiring *S aureus* infection during hospitalization.

**Meaning:**

These findings suggest that nasal colonization is a relevant risk factor for MSSA infection in a nonoutbreak neonatal intensive care unit setting.

## Teaser

This cohort study analyzes methicillin-susceptible *Staphylococcus aureus* (MSSA) colonization and infection events among patients in a single neonatal intensive care unit (NICU) and assesses the performance of *spa* typing and whole-genome sequencing for routine *S aureus* surveillance.

## Introduction

Preterm newborns, especially those with low to very low birth weight, are vulnerable to nosocomial infections, such as those caused by *Staphylococcus aureus*. Indeed, *S aureus* infections are among the leading causes of health care–associated infections in neonatal intensive care units (NICUs).^1,2,3^ However, most studies focus on methicillin-resistant *S aureus* (MRSA), although infections with antibiotic-susceptible *S aureus* might be even more important in terms of morbidity and mortality, considering their reportedly higher prevalence when compared with MRSA infections.^2,4,5^

Colonization with *S aureus*, in addition to extended hospital stay and low birth weight, is considered a significant risk factor for acquiring *S aureus* infections in NICUs.^6^ Therefore, prevention of transmission and eradication of colonization is thought to be beneficial in reducing infections.^7,8,9,10^ The colonization and transmission dynamics in newborns in a nonoutbreak setting are not yet fully understood, because most studies focus on transmissions in outbreak settings.^1,11^ Commonly, colonization occurs in the postnatal phase and may be acquired through vertical transmission from mother to infant, or from health care workers (HCWs), parents, visitors, or the environment to infant.^12,13,14^

In this study, we performed a retrospective analysis of all *S aureus* colonization and infection events during a 2-year period in the NICU of our tertiary hospital. Data from the weekly screenings for *S aureus* in all newborns admitted to the NICU were used to improve our understanding of the interplay between colonization and transmission. In addition, we assessed the performance of staphylococcal protein A (*spa*) typing using the polymorphic gene region encoding protein A as our standard typing method with whole-genome sequencing (WGS) to evaluate the latter’s added benefit for routine surveillance of *S aureus* in a given setting.

## Methods

### Study Premise

In this cohort study, we enrolled all premature newborns admitted to the NICU of the Heidelberg University Hospital from January 1, 2018, to December 31, 2019. The NICU consists of 22 stationary beds that are assembled as two 4-bed rooms and six 2-bed rooms. The primary end point of this observational cohort study was *S aureus* infection during hospitalization with *S aureus* colonization as exposure. Secondary outcomes were transmission of *S aureus* in a nonoutbreak setting and the concordance of *spa* typing and WGS for routine surveillance and outbreak detection. Weekly screenings of newborns were conducted as part of the local infection control and screening policy for multidrug-resistant organisms as implemented by the Department of Medical Microbiology and Hygiene of Heidelberg University Hospital. The local ethics committee was consulted before the study begin and waived individual informed consent owing to deidentified data. This study adhered to the Strengthening the Reporting of Observational Studies in Epidemiology (STROBE) reporting guideline.

### Routine Screening and Infection Control Measures

Screening for *S aureus* colonization was performed weekly in the postnatal period, together with screening for cephalosporin- and carbapenem-resistant gram-negative bacteria from the nasal (anterior nares) and rectal/perianal cavities using 2 separate swabs, 1 for each site.

Basic hygiene measures were applied for any patient contact, including patients colonized with methicillin-susceptible *S aureus* (MSSA). These measures included consistent hand disinfection in accordance with the 5 World Health Organization indications and the wearing of disposable gloves and protective gowns to avoid contamination of staff where direct contact with blood, secretions, excrement, mucous membranes, or nonintact skin is expected.

Furthermore, the following infection prevention and control measures were implemented: (1) disposable apron for nursing rounds and in case of contamination risk of the body front; (2) protective gown, if the child is carried; (3) regular and hygiene training and compliance observations (at least once a year), on-site visits (at least once a year), and quality meetings with the infection control team (at least 3 times a year); (4) 24-hour monitoring of automated hand disinfectant use at every bed site; (5) surgical face mask for the care of patients with MSSA or MRSA to avoid droplet contamination; (6) isolation room and contact precautions for patients with MRSA or cephalosporin- and carbapenem-resistant gram-negative bacteria; and (7) decolonization of patients with MRSA using a 5-day scheme, including antiseptic nasal, oral, and skin treatment. No routine decolonization measures were implemented for MSSA.

### Laboratory Methods

Routine screening samples were processed in the microbiological diagnostics laboratory of the university hospital using the total laboratory automation system (Kiestra; Becton and Dickinson) as published elsewhere, with minor modifications.^15,16^ Briefly, swabs were inoculated onto a biplate chromogenic medium for *S aureus* and MRSA detection (CHROMagar Staph aureus/MRSAII; BD Diagnostics) and Columbia blood agar with 5% sheep blood as a growth control for sampling validity. Species identification was performed via matrix-assisted laser desorption ionization (MALDI-TOF; Bruker) using a score of at least 2.0 as a cutoff. Antibiotic susceptibility testing was not performed routinely for *S aureus* isolates from screening samples. The *S aureus* isolates were cryopreserved at −70°C for molecular typing.

### Molecular Typing

Crude bacterial DNA was extracted using a rapid extraction method with lysostaphin (Genaxxon Bioscience) and proteinase K (Bio&Sell) as described previously.^17^ Conventional routine *spa* typing was performed as described elsewhere^18^; *spa* gene sequences were analyzed using Ridom StaphType software, version 2.2.1 (Ridom GmbH). Data on global frequency were obtained through the Ridom SpaServer website.^19^

### WGS and Data Analysis

Genomic DNA for WGS was extracted from overnight bacterial culture using a blood-typing kit (DNeasy Blood and Tissue Minikit; Qiagen GmbH) according to the manufacturer’s protocol, with the addition of an initial lysis step with lysostaphin. Genomic DNA was sequenced (MiSeq system; Illumina, Inc; 2 × 250 base pairs [bp] paired-end) as described previously.^16^

Raw sequences were trimmed for quality using Sickle, version 1.33 (parameters, q > 30; l > 45).^20^ The cleaned sequences were then assembled using SPAdes, version 3.13.0.^21^ Assembled contigs were curated for length (>500 bp) and coverage (>×10). Annotation was performed using Prokka, version 1.14.1 (based on Genetic Code Table 11)^22^ and the National Center for Biotechnology Information (NCBI) Prokaryotic Genome Annotation Pipeline. Resistance and virulence genes were found using ABRicate, version 0.8.13^23^ with antibiotic databases from ResFinder 3.0 (Antibiotic Resistance Gene–Annotation [ARG-ANNOT]; Comprehensive Antibiotic Resistance Database [CARD]; NCBI–Bacterial Antimicrobial Resistance Reference Gene Database [NCBI-BARRGD]; and the Virulence Factor Database [VFDB]). Multilocus sequence type (MLST) was derived from the draft genome using the MLST 2.0 pipeline from the Center for Genomic Epidemiology.^24^ Sequences were deposited to the NCBI GenBank under the bioproject number PRJNA637212. Accession numbers as well as sequencing statistics are provided in eTable 1 in the Supplement.

Genome comparison was performed using the core genomes with Roary (1720 genes; mean [SD], 66.6% [1.9%] of the genomes).^25^ Phylogenetic distance was calculated with Gubbins, version 2.3.4, to consider recombination events and not overestimate the single-nucleotide variant (SNV).^26^ In total, 69 323 polymorphic sites were kept (hqSNV) and clonal groups were defined as genomes distant from less than 20 SNVs (0.03% of the polymorphic sites as suggested by Cremers et al).^11^ Minimum spanning tree was calculated using MSTree, version 2, and visualized using Grapetree.^27^

### Statistical Analysis

Appropriate measures of the location and spread of the distribution of sociodemographic and clinical variables were tabulated by *S aureus* colonization or infection status. We used univariable and multivariable logistic regression to estimate the change in the odds of *S aureus* colonization and infection in the presence of putative risk factors of these outcomes, together with their 95% CIs, and tested against the null hypothesis (H_0_) with an odds ratio [OR] of 1.00 using an α of .05. Test of the collinearity for correlations of the variables was performed using the (variance inflation factor) command following a regression model with all relevant variables. The mean of the variance inflation factor was 1.41 (range, 1.09-1.89), indicating no collinearity between variables. All statistics were performed in STATA, version 13 (StataCorp LLC).

## Results

A total of 590 newborns treated in the NICU during the 2-year study period were included in this study, of whom 276 (46.8%) were female and 314 (53.2%) were male; 477 (80.9%) were preterm newborns; 449 (76.1%) were singletons; and 220 (37.3%) had a birthweight of less than 1500 g. Of 586 newborns with delivery mode data, 419 (71.5%) were delivered via cesarean section and 167 (28.5%) via natural vaginal delivery. Patient demographics are summarized in Table 1. Forty-eight sets of twins and 5 sets of triplets were included in the study population. Overall, 135 newborns (22.9%) had *S aureus* colonization in the nasal cavity and/or rectum during the course of their hospital stay. Of these, 81 newborns (60.0%) had exclusive colonization in the nose, 5 (3.7%) had exclusive rectal colonization, and 49 (36.3%) had both nasal and rectal colonization. Only 1 newborn had MRSA colonization. Of 135 infants with colonization, 32 (23.7%) acquired colonization within the first 7 days after delivery and 97 (67.4%) by the end of the first month of life.

**Table 1.** Clinical Characteristics of Study Infants

Characteristic	Newborn group^a^			Crude analysis^b^		Adjusted^c^	
	All (N = 590)	Colonization (n = 135)	Noncolonization (n = 455)	OR (95% CI)	*P* value	OR (95% CI)	*P* value
Sex							
Female	276 (46.8)	66 (48.9)	210 (46.2)	1.1 (0.8-1.6)	.60	NA	NA
Male	314 (53.2)	69 (51.1)	245 (53.8)	1 [Reference]	NA	NA	1 [Reference]
Birthweight <1500 g^d^	220 (37.3)	103 (76.3)	117 (25.7)	9.3 (5.9-14.6)	<.001	3.6 (1.9-6.6)	<.001
Preterm	477 (80.8)	125 (92.6)	352 (77.4)	3.7 (1.9-7.2)	<.001	0.8 (0.4-1.8)	.60
Multiple gestation	141 (23.9)	41 (30.4)	100 (22.0)	1.5 (1.0-2.4)	.05	1 (0.6-1.7)	.96
Delivery mode^d^							
Cesarean section	419 (71.5)	115 (85.2)	304 (67.4)	2.8 (1.7-4.7)	<.001	1.8 (1.0-3.4)	.05
Vaginal	167 (28.5)	20 (14.8)	147 (32.6)	1 [Reference]	NA	NA	1 [Reference]
Length of stay, median (IQR), d	26 (10-62)	69 (44-104)	19 (8-41)	2.3 (1.9-2.7)^e^	<.001	1.7 (1.4-2.1)	<.001
Colonization site							
Nasal	NA	81 (60.0)	NA	NA	NA	NA	NA
Rectal	NA	5 (3.7)	NA	NA	NA	NA	NA
Nasal and rectal	NA	49 (36.3)	NA	NA	NA	NA	NA
*S aureus* infections							
Any	10 (1.7)	7 (5.2)	3 (0.7)	NA	NA	NA	NA
BSI	6 (1.0)	3 (2.2)	3 (0.7)	NA	NA	NA	NA
Other^f^	4 (0.7)	4 (3.0)	0	NA	NA	NA	NA

Abbreviations: BSI, bloodstream infection; IQR, interquartile range; NA, not applicable; OR, odds ratio.

^a^ Unless otherwise indicated, data are expressed as number (%) of patients.

^b^ Ratio of odds of nasal colonization, calculated using a univariate logistic regression model.

^c^ Ratio of odds of nasal colonization, calculated using a multivariate logistic regression model, with birth weight, gestational age, multiple gestation, delivery mode, and length of stay (per 30 days). Mean variance inflation factor was 1.41 (range, 1.09-1.89), indicating no collinearity.

^d^ Four values were missing for newborns without colonization.

^e^ OR expressing increase in odds of colonization per 30 days increase in length of hospitalization.

^f^ Other infections included conjunctivitis (n = 2) and skin and soft tissue infections (n = 2).

### Risk Factors for *S aureus* Infections

Ten of 590 infants (1.7%; 0.41 infections per 1000 patient-days) had *S aureus* infections during their stay in the NICU. Six of 10 infections (6 of 590 [1.0% overall incidence]; 0.25 infections per 1000 patient-days) were bloodstream infections, 2 were skin and soft tissue infection (abscess), and 2 were *S aureus* conjunctivitis. Colonization with *S aureus* was significantly associated with the acquisition of any *S aureus* infection (OR, 8.2; 95% CI, 2.1-32.3; *P* = .002) (Table 2). We also observed a trend in which carriers were more likely to acquire bloodstream infection (OR, 3.4; 95% CI, 0.7-17.2; *P* = .10) than newborns without colonization. Of the 10 infections, 7 were colonized. Four of the 7 (57.1%) infection strains were recoverable for sequencing and all of them were genetically identical to the colonization strain (<20 hqSNVs between infection and colonization isolates); thus, endogenous infection was likely. All infection strains were genetically unrelated.

**Table 2.** Risk Factors for *Staphylococcus aureus* Infections in Hospitalized Infants in the Neonatal Intensive Care Unit

Risk factor	Newborn group^a^			Crude analysis	
	All (N = 590)	*S aureus infection* (n = 10)^b^	No *S aureus* infection (n = 580)	OR (95% CI)	*P* value
*S aureus* colonization	135 (22.9)	7 (5.2)	128 (94.8)	8.2 (2.1-32.3)	.002
Female	276 (46.8)	7 (2.5)	269 (97.5)	2.7 (0.7-10.5)	.20
Birthweight <1500 g^c^	220 (37.3)	6 (2.7)	214 (97.3)	2.6 (0.7-9.2)	.10
Preterm (gestational age <37 wk)^c^	477 (80.8)	9 (1.9)	468 (98.1)	2.2 (0.3-17.2)	.50
Multiple gestation	141 (23.9)	3 (2.1)	138 (97.9)	1.4 (0.4-5.4)	.60
Cesarean section delivery^c^	419 (71.0)	9 (2.1)	410 (97.9)	3.7 (0.5-29.3)	.20
Length of stay, median (IQR), d	26 (10-62)	59 (50-138)	25.5 (10-61)	1.5 (1.1-1.9)	.003

Abbreviations: IQR, interquartile range; OR, odds ratio.

^a^ Unless otherwise indicated, data are expressed as number (%) of all newborns; for subgroups, number (%) of row total.

^b^ Infection rate is 0.41 for any *S aureus* infection per 1000 patient-days and 0.25 per 1000 patient-days for *S aureus* bloodstream infections (OR, 3.4; 95% CI, 0.7-17.2; *P* = .10 for *S aureus* bloodstream infection).

^c^ Two values were missing for birthweight and gestational age; 4 values were missing for delivery mode.

### Risk Factors for *S aureus* Colonization

**Figure 1.**
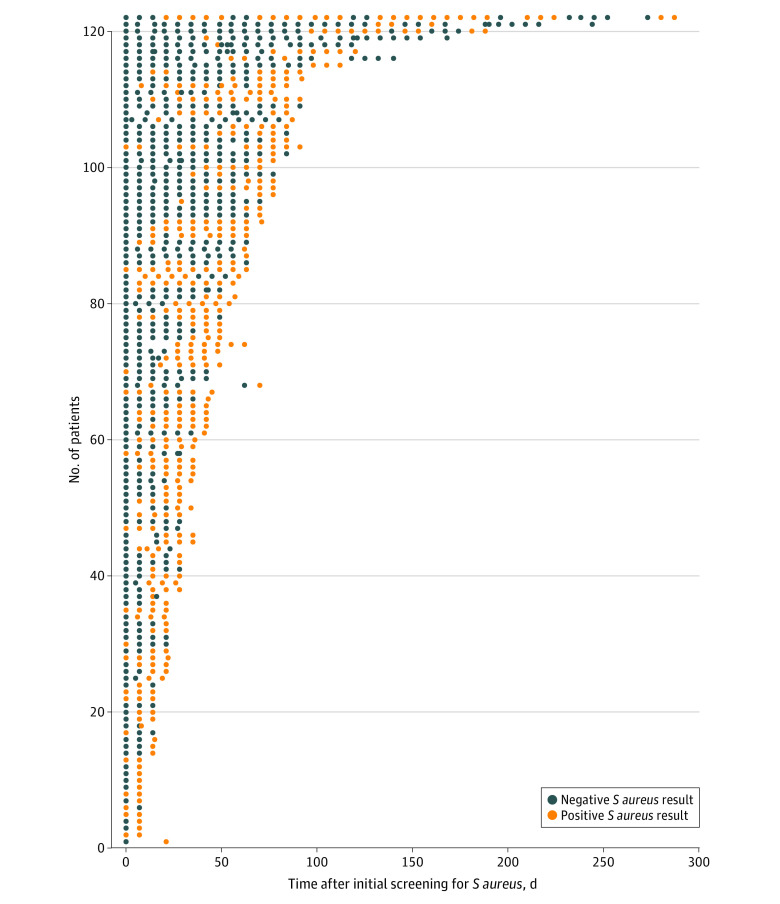
*Staphylococcus aureus* Colonization Dynamics in Newborns Admitted to the Neonatal Intensive Care Unit Over Time We observed 2 different patterns of colonization: persistent (positive subsequent screening after initial *S aureus* detection) and transient. Only patients with more than 1 swab were included in the analysis.

The median length of NICU stay was 26 (interquartile range [IQR], 10-62) days. The median time to first detection (birth to first positive nasal/rectal swab finding) was 17 (IQR, 11-37) days. Colonization with *S aureus* was associated with low birth weight (<1500 g) and longer median length of stay (69 [IQR, 44-104] vs 19 [IQR, 8-41] days). Surgical delivery was also associated with *S aureus* colonization (Table 1). One hundred twenty-three of 135 nasal *S aureus* isolates (91.1%) were available for sequencing (12 were nonrecoverable). The colonization dynamics summarized in Figure 1 suggest 2 colonization patterns: persistent (subsequent positive screening result after initial *S aureus* detection until discharge) and transient (intermittent positivity).

### Molecular Characteristics

The most prevalent (≥10 isolates per cluster) MLST of the 123 sequenced *S aureus* isolates were ST45, ST30, ST15, and ST121. The most prevalent *spa* types, with more than 5 isolates each in the study population, were t084, t170, t2642, and t571. An overview of all *spa* types is summarized in eTable 2 in the Supplement. Panton-Valentine leukocidin was detected in 2 methicillin-susceptible isolates (ST152 t084 and ST34 t1758). One MRSA isolate belonged to ST225 t003 with SCC*mec* type II.

### Transmission Clusters

Altogether, 123 colonization isolates were characterized by *spa* and WGS. Using *spa* types to identify potential transmissions, we obtained 27 potential events (≥2 patients with identical *spa* types) involving 95 patients (eTable 2 in the Supplement). More than one-third (10 of 27 [37.0%]) of the transmission clusters involved 4 or more patients. The number of patients involved in each *spa* transmission cluster is displayed in eTable 2 in the Supplement.

**Figure 2.**
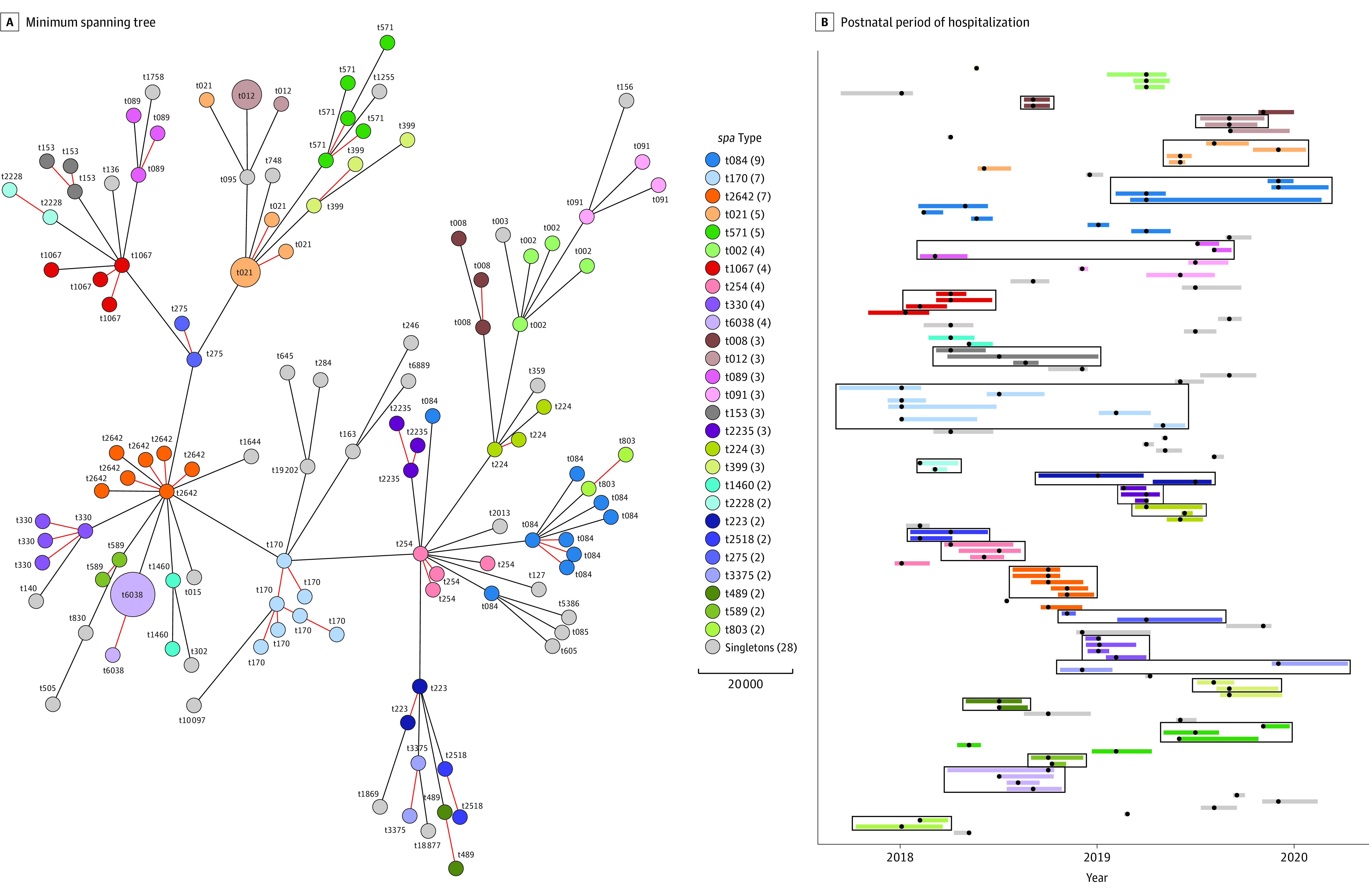
Minimum Spanning Tree and Gantt Diagram for Epidemiological Overlap Within the Potential Transmission Clusters A, Minimum spanning tree of all colonization isolates in the study, based on 1720 core genomes (mean [SD], 66.6% [1.9%] of the genomes). Node size indicates the number of isolates with identical core genome (0 single nucleotide variants [SNVs]). Genetically closely related isolates (SNV <24) are indicated by red branches/connecting lines. B, Visualization of the postnatal period of hospitalization. Black dots indicate the first detection of colonization. Isolates with close genetic relationship (transmission clusters) are indicated with squares. Nodes (in part A) and bars (in part B) were colored according to the sequencing of the polymorphic gene region encoding staphylococcal protein A (*spa*) type.

In comparison, using a minimum spanning tree based on the hqSNVs to depict the phylogenetic relatedness among all colonization isolates (n = 123), we obtained 23 potential transmission clusters, of which only 6 (26.1%) involved 4 or more patients in the respective transmission groups (Figure 2). Compared with *spa* typing (95 patients), WGS suggests that only 70 patients were involved in the transmission clusters, which indicates an overestimation of transmission events by molecular typing based on *spa* type only. Discordance was mainly observed for *spa* types with high global frequency in the publicly available Ridom *spa* database (eFigure in the Supplement). In contrast, low-frequency *spa* types correlate with the epidemiological overlap and WGS transmission clusters (Figure 2 and eFigure in the Supplement). For example, despite the suspicious epidemiological overlap in clinic stay for the t002 *spa* transmission cluster (Figure 2B), SNV analysis did not indicate a close genetic relationship (48-334 SNVs between isolates). Indeed, *spa* type t002, with more than 6.5% global frequency, belongs to the more common *spa* types; therefore, the accumulation of *spa* type t002 is not a reliable indicator for transmission events. Most WGS transmission clusters displayed plausible temporal-spatial overlap. However, for 2 clusters, t089 and t3375 (Figure 2B), there was a gap of several months between detection of new cases in the transmission cluster, which may be an indication of an external reservoir.

### *S aureus* Colonization in Twins and Triplets

**Figure 3.**
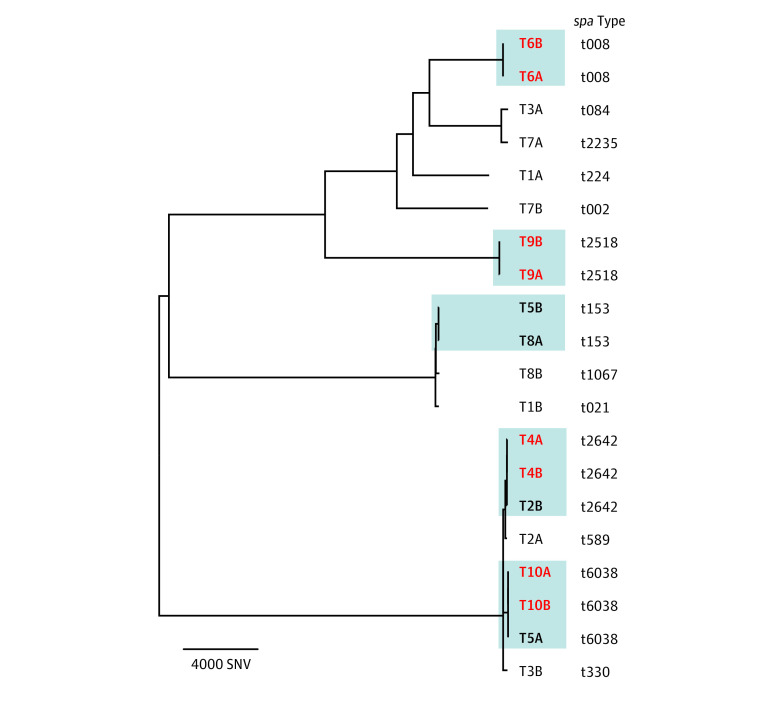
Genetic Relatedness of *Staphylococcus aureus* Isolates From Twins With Colonization Twins with identical *S aureus* clones are indicated by bold red font. Isolates belonging to a potential transmission cluster (ie, genetically closely related), according to the defined high-quality single-nucleotide variant (SNV) cutoff (<20), are indicated by gray shading. Only 4 of 10 (40.0%) twins had colonization by identical clones. There were 4 potential interfamilial transmissions, indicated by bold black font within the gray shading. *spa *indicates staphylococcal protein A.

In 12 of the 48 sets of twins in our study cohort (25.0%), both newborns had *S aureus* colonization. In 13 of 48 sets (27.1%), the colonization status was discordant (ie, only 1 twin had colonization). In 23 of 48 sets of twins (47.9%), neither infant had colonization. All 5 sets of triplets had no colonization during hospitalization (Table 1). Colonization isolates from 10 twin pairs were available for WGS (Figure 3). The isolates in 4 twin pairs (40.0%; T4, T6, T9 and T10) were genetically identical (hqSNV <20) (Figure 3). Three suspected interfamilial transmission clusters (T5B-T8A, T4A-T4B-T2B, and T10A-T10B-T5A) had an epidemiological link (Figure 3).

## Discussion

In our study, most newborns admitted to the NICU were premature (delivery at gestational age of <37 weeks and birthweight of ≤1500 g). Premature newborns generally require longer NICU stays,^28^ providing ample opportunities to acquire *S aureus* colonization during hospitalization. Overall, 22.9% of the newborns in our study population were colonized by *S aureus*, which is consistent with the expected general colonization proportion (20%-30%) in the population. Thus, these findings may represent a physiological process instead of a pathological phenomenon.^29,30,31^ A significant percentage of these infants (23.7%) acquired colonization in the first week after delivery, and 71.9% had a positive in *S aureus* screening result within their first month of life. Nasal colonization appears to be the primary and most important reservoir in this population, because only 3.7% of the colonized infants were exclusively colonized in the perianal/rectal region. Our findings are consistent with published data on the colonization sites of infants in the NICU setting.^11,32^

Overall, the acquisition of *S aureus* infection was a rare event. Only 1.0% and 1.7% of all NICU patients in our study acquired *S aureus* bloodstream infections or any type of *S aureus* infections, respectively. Our data imply that hospitalized infants with *S aureus* colonization at the NICU develop *S aureus* infections more frequently than those without colonization, independent of their length of stay. All our *S aureus* infections in carriers were endogenous; that is, colonization and infection strains were clonally identical. Indeed, colonization with *S aureus*, regardless of methicillin resistance, has frequently been described as one of the major risk factors for the acquisition of *S aureus* infections, both in adult and pediatric populations.^1,33,34^ Length of stay in the NICU has been reported as one of the contributing factors for *S aureus* infections,^6,35^ which can be influenced by birth weight, gestational age, and other clinical parameters.^36^ Because these parameters are only marginally modifiable, decolonization measures have been suggested as an option to prevent *S aureus* infections in this population.^8^

Multiple possible routes of acquisition for *S aureus* colonization exist in newborns. Apart from parent-infant transmission, deficits in hygiene measures, environmental contamination, and transmissions mediated by HCWs and visitors are plausible.^13,37,38^ Whole-genome sequencing together with the epidemiological data indicated that there were 23 transmission clusters, with more than one-quarter of these clusters involving 4 or more patients. Hence, transmission events occur frequently, and routine surveillance is necessary to monitor and contain transmission chains and outbreaks at an early point. We observed 2 distinct transmission cluster patterns: (1) classical transmission events with clear genetic and epidemiological overlap and (2) interrupted transmission events, with genetic concordance but significant time lapse between new detections. The latter may indicate the presence of a potential environmental reservoir (eg, HCWs and contaminated inanimate objects) in the ward.

Parent-infant transmission is regarded as one of the most probable acquisition routes for *S aureus* colonization in newborns.^14,39,40^ Although mother-infant transmission is plausible, published data on mother-infant transmissions are not consistent. A study by Leshem et al^40^ reported a high genetic concordance of 80% between mother-infant *S aureus* strains, whereas a study in an African setting^39^ reported low rates of mother-infant transmissions. Owing to the study setting, we cannot assess the role of mother-infant transmissions in our study, because *S aureus* screening of the parents was only performed sporadically and thus cannot be included in the analysis. However, if parent-infant transmission were the route of acquisition, one would expect that siblings would share identical clones, because parents only had contact with their own newborns during skin-to-skin care.^13^ The fact that genetically closely related clones were detected in unrelated newborns indicates a different acquisition route.

The genetic and epidemiological link suggests that patient-to-patient transmission via hands of HCWs could contribute to the acquisition of colonization in newborns in the NICU.^2,37^ In contrast to adult patients, these newborns are not mobile and are confined within their own incubators, so that direct newborn-to-newborn contact and transmission would have to be mediated by mobile vectors. Indeed, the role of HCWs as vectors for transmission has been reported numerously.^11,37,38^ Because mandatory screening of HCWs in Germany requires specific approval and compliance, which would only be granted for outbreak investigations, we cannot incorporate *S aureus* from HCWs in our study. Nevertheless, molecular typing, along with epidemiological patient data and transmission patterns, suggests the involvement of HCWs as a transmission vector and warrants further scrutiny.

High discriminatory molecular typing such as WGS can be costly and may not be readily accessible. The *spa* typing method seems to be reliable and affordable for surveillance, especially when interpreted in conjunction with the global *spa* type frequency. This method will probably exaggerate the magnitude of an outbreak owing to the limited discriminatory power compared with WGS,^41^ but most importantly, *spa* typing did not produce very major errors (ie, false-negative findings).

### Limitations

Our study has some limitations. This was a single-center study, so generalization of the findings may be limited. The incidence rates of infections differ significantly between hospitals, and the hygiene standards and measures are not harmonized on a national level. Nevertheless, the large sample size allows us to investigate the transmission of *S aureus* in a nonoutbreak NICU setting. Although the sample size may be adequate to study transmissions within in the hospital setting, the sample size may be too small to accurately assess the incidence of infection events, and thus the incidence may be underestimated or overestimated. Numerous studies on the risk factors for *S aureus* infections in patients in the NICU were performed in outbreak settings and focused mainly on MRSA; therefore, the relevance and impact of the risk factors may have been exaggerated against an elevated incidence for colonization and infection owing to large outbreak clusters. Furthermore, our NICU has only shared rooms (2-bed or 4-bed), so the impact of private rooms or preemptive isolation cannot be assessed in our study setting.

## Conclusion

This cohort study found that the incidence of *S aureus* infection was very low in a nonoutbreak NICU setting. Our data suggest that nasal colonization is a relevant risk factor for *S aureus* infection in neonates and that minor transmission events occur in a nonoutbreak setting. Furthermore, molecular characterization suggested the presence of transmission vectors or source in facilitating the acquisition and spread of *S aureus* colonization in premature newborns in a NICU setting, which warrants further investigation and validation.

## Notes

Supplement. **eFigure.** Correlation Between Global Frequency of *spa* Types and WGS Transmission Clusters **eTable 1.** Sequencing Statistics **eTable 2.***spa* Type Frequency
