# Supplementary material for: Surveillance for Colonization, Transmission, and Infection With Methicillin-Susceptible Staphylococcus aureus in a Neonatal Intensive Care Unit
Source: JAMA Netw Open. 2021 Sep 13;4(9):e2124938. doi: 10.1001/jamanetworkopen.2021.24938 (PMC8438598; doi:10.1001/jamanetworkopen.2021.24938)
Supplement: Supplement. — eFigure. Correlation Between Global Frequency of spa Types and WGS Transmission Clusters eTable 1. Sequencing Statistics eTable 2.spa Type Frequency [file jamanetwopen-e2124938-s001.pdf]

Supplementary Online Content

Nurjadi D, Eichel VM, Tabatabai P, et al. Surveillance for colonization, transmission, and infection with methicillin-susceptible *Staphylococcus aureus* in a neonatal intensive care unit. *JAMA Netw Open*. 2021;4(9):e2124938. doi:10.1001/jamanetworkopen.2021.24938

eFigure. Correlation Between Global Frequency of *spa* Types and WGS Transmission Clusters
eTable 1. Sequencing Statistics
eTable 2. *spa* Type Frequency

This supplementary material has been provided by the authors to give readers additional information about their work.

© 2021 Nurjadi D et al. *JAMA Network Open*.

eFigure. Correlation Between Global Frequency of *spa* Types and WGS Transmission Clusters

The *spa*-types in the y-axis are sorted by the global frequency (highest to lowest), data from Ridom Staphtype Database. The size of the circles corresponds to the number of isolate within the *spa*-type group. The x-axis indicates the percentage (%) of the *spa*-types involved in a transmission cluster, as defined by the high-quality SNP cut-off (<20) for transmission events.

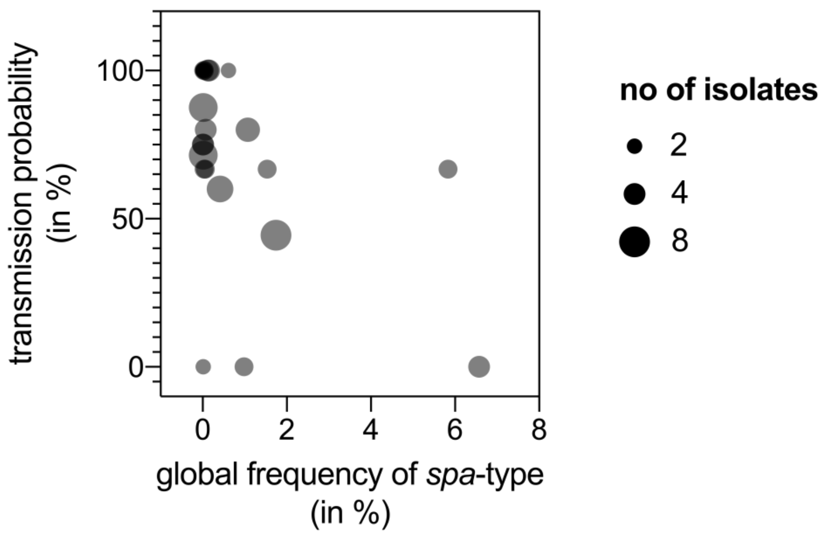

© 2021 Nurjadi D et al. *JAMA Network Open*.

eTable 1. Sequencing Statistics

All sequences were uploaded to the NCBI Genbank under the bioproject number PRJNA637212

sample_name	Accession	coverage	# contigs	Largest contig	Total length	GC (%)	N50	N75	L50	L75
BK30250	SAMN18024830	70	12	1295038	2661434	32.77	1028159	1028159	2	2
BK30794	SAMN18024831	50	19	977507	2802638	32.64	752469	172736	2	4
BK30893	SAMN18024832	48	35	239220	2805222	32.72	131045	92810	9	15
BK31015	SAMN18024833	50	30	371244	2748536	32.72	152172	87805	6	12
D1907	SAMN18024834	24	45	368357	2761932	32.8	131918	80293	8	15
D2068	SAMN18024835	42	24	937718	2765443	32.8	495696	124766	2	5
D2163	SAMN18024836	21	61	371082	2742249	32.85	93720	41038	9	21
D2212	SAMN18024837	26	36	621538	2756117	32.81	209881	106523	4	8
D2235	SAMN18024838	107	18	1006257	2766261	32.79	620297	187950	2	4
D2254	SAMN18024839	84	19	989839	2766212	32.79	620297	187950	2	4
D2263	SAMN18024840	89	18	989839	2765825	32.79	620207	184316	2	4
KE5608	SAMN18024841	43	28	371244	2748912	32.73	155036	87806	6	12
KE5799	SAMN18024842	34	17	625275	2870441	32.72	456965	243829	3	5
KE5911	SAMN18024843	38	43	488492	2847991	32.69	127776	79888	7	14
KE5912	SAMN18024844	36	28	371244	2749036	32.73	210360	92697	5	11
KE6081	SAMN18024845	34	24	366522	2710861	32.71	203828	96929	5	9
KE6378	SAMN18024846	32	16	547503	2665318	32.69	252596	244692	4	6
KE6527	SAMN18024847	38	31	435476	2726982	32.76	162912	99546	5	10
KE6528	SAMN18024848	42	47	509512	2846195	32.72	133619	71207	7	14
KE6529	SAMN18024849	42	43	488492	2848020	32.69	127776	79888	7	14
KE6598	SAMN18024850	37	15	566425	2727161	32.69	434612	232567	3	5
KE6600	SAMN18024851	37	38	280544	2741029	32.72	130907	92812	8	14
KE6602	SAMN18024852	50	18	547731	2665742	32.7	252379	244692	4	6
KE6603	SAMN18024853	38	30	371244	2753584	32.72	152115	87806	6	12
KE6628	SAMN18024854	52	48	509512	2845223	32.72	134731	71207	7	14
KE6692	SAMN18024855	38	28	371244	2748670	32.72	154794	87806	6	12
KE6736	SAMN18024856	49	31	644839	2775110	32.7	182403	134996	5	9
KE6744	SAMN18024857	41	39	280544	2742056	32.72	130907	90157	8	14
KE6746	SAMN18024858	41	19	765166	2665928	32.79	378823	343980	3	4
KE6801	SAMN18024859	44	33	547616	2775193	32.7	181622	97013	5	10
KE6808	SAMN18024860	42	42	488492	2847531	32.69	127776	79888	7	14
KE6810	SAMN18024861	41	41	488305	2846527	32.69	127776	79888	7	14
KE6877	SAMN18024862	32	51	309445	2874501	32.7	150429	67353	8	14
KE6928	SAMN18024863	44	36	297894	2741918	32.72	161494	92812	7	12
KE6951	SAMN18024864	41	8	1224708	2739684	32.69	747337	314917	2	3
KE7007	SAMN18024865	34	36	272322	2864779	32.71	150491	87823	8	14
KE7022	SAMN18024866	41	17	1026485	2737843	32.79	789194	174749	2	4
KE7129	SAMN18024867	47	17	758487	2756527	32.78	620793	267591	2	5
KE7242	SAMN18024868	40	19	536253	2726183	32.81	409254	301421	3	5
KE7246	SAMN18024869	41	24	393316	2755463	32.74	243823	154765	5	8
KE7406	SAMN18024870	53	17	568488	2665957	32.8	533532	378822	3	4
KE7407	SAMN18024871	38	18	475569	2708842	32.69	317373	206845	4	7
KE7408	SAMN18024872	32	24	367474	2713279	32.71	203802	123682	5	9
KE7415	SAMN18024873	42	23	570185	2711254	32.71	201142	96929	4	9
KE7476	SAMN18024874	41	17	580824	2760720	32.73	437241	252242	3	5
KE7512	SAMN18024875	50	23	367474	2713053	32.71	201142	96929	5	10
KE7635	SAMN18024876	54	39	401462	2809844	32.71	175023	76456	6	13
KE7757	SAMN18024877	40	15	1342704	2816667	32.77	630720	392447	2	3
KE7780	SAMN18024878	40	53	267651	2788316	32.8	80990	45049	10	21
KE7833	SAMN18024879	51	30	371243	2749437	32.73	155035	87805	6	12
KE7881	SAMN18024880	42	53	267651	2786773	32.8	92882	46216	10	21
KE7882	SAMN18024881	44	14	1342812	2825188	32.77	630720	392447	2	3
KE7884	SAMN18024882	65	37	422039	2807497	32.71	162916	96434	6	11
KE7950	SAMN18024883	44	12	1342812	2816705	32.77	630720	392447	2	3
KE7977	SAMN18024884	37	35	272322	2864035	32.71	150491	87825	8	14
KE7978	SAMN18024885	43	13	1342812	2818086	32.78	630720	392447	2	3
KE8017	SAMN18024886	44	36	272322	2864216	32.71	150493	87825	8	14

© 2021 Nurjadi D et al. *JAMA Network Open*.

KE8135	SAMN18024887	38	15	865725	2732474	32.69	589789	401884	2	4
KE8223	SAMN18024888	43	16	1165892	2730262	32.69	589789	380226	2	3
KE8262	SAMN18024889	44	17	1220203	2715814	32.82	604128	399415	2	3
KE8398	SAMN18024890	36	10	1298307	2689302	32.83	622124	404179	2	3
KE8477	SAMN18024891	46	27	458349	2759380	32.69	173988	112333	5	10
KE8480	SAMN18024892	40	27	457713	2762147	32.68	176739	126971	5	9
KE8525	SAMN18024893	43	12	1193894	2690720	32.83	622124	404179	2	3
KE8601	SAMN18024894	36	37	419988	2841265	32.72	154705	96486	5	10
KE8759	SAMN18024895	36	13	1190602	2661187	32.77	1028159	1028159	2	2
KE8760	SAMN18024896	42	16	660886	2756440	32.73	582292	314626	3	4
KE8762	SAMN18024897	42	12	814485	2715392	32.72	707338	233219	2	4
KE8783	SAMN18024898	38	18	985273	2743879	32.79	422495	338571	2	4
KE8872	SAMN18024899	39	18	1028875	2746629	32.77	748497	526025	2	3
KE8962	SAMN18024900	37	18	561069	2669695	32.69	318844	243886	3	6
KE8964	SAMN18024901	45	19	1028876	2746843	32.77	748389	399675	2	3
KE8969	SAMN18024902	101	18	564471	2677900	32.82	230375	167920	4	7
KE9018	SAMN18024903	41	19	1028875	2746966	32.77	748497	399676	2	3
KE9019	SAMN18024904	41	27	371244	2748684	32.73	210282	92697	5	11
KE9100	SAMN18024905	40	19	1028314	2746512	32.77	748497	399675	2	3
KE9232	SAMN18024906	41	19	660592	2719738	32.69	249696	191968	4	7
KE9243	SAMN18024907	77	15	459100	2717093	32.73	319129	249958	4	6
KE9287	SAMN18024908	26	33	384397	2776389	32.74	185279	127911	6	10
KE9419	SAMN18024909	85	20	547416	2732787	32.83	410732	211862	3	6
MR100	SAMN18024910	36	28	371244	2748481	32.72	154794	87805	6	12
MR101	SAMN18024911	28	20	707890	2741411	32.75	325103	163254	3	5
MR105	SAMN18024912	33	29	451854	2788800	32.74	197723	92781	5	11
MR112	SAMN18024913	34	40	323681	2760301	32.74	174043	83869	6	12
MR119	SAMN18024914	37	14	769676	2776976	32.72	706683	194930	2	4
MR120	SAMN18024915	31	21	517966	2801022	32.67	329449	106264	4	7
MR121	SAMN18024916	30	41	321836	2812037	32.72	150857	64346	7	13
MR122	SAMN18024917	29	36	419988	2839235	32.72	154705	94561	5	10
MR135	SAMN18024918	28	27	406483	2726351	32.71	173988	112333	6	11
MR137	SAMN18024919	88	21	547416	2732811	32.83	410732	211889	3	6
MR146	SAMN18024920	30	40	321836	2811936	32.72	150892	64346	7	13
MR154	SAMN18024921	34	16	796115	2735337	32.69	672424	168988	2	5
MR156	SAMN18024922	31	14	1108564	2689218	32.8	423006	214385	2	4
MR170	SAMN18024923	30	14	797075	2734256	32.68	672423	187128	2	5
MR171	SAMN18024924	26	17	611285	2718761	32.7	318608	249961	3	6
MR206	SAMN18024925	30	16	796115	2735529	32.69	672423	168988	2	5
MR207	SAMN18024926	23	37	379319	2803758	32.71	150913	85259	6	12
MR234	SAMN18024927	26	42	509368	2836320	32.65	133618	78820	7	14
MR236	SAMN18024928	29	12	703658	2710993	32.72	656000	319407	2	4
MR257	SAMN18024929	65	37	298962	2804603	32.72	150916	76454	7	13
MR262	SAMN18024930	70	36	350873	2804802	32.72	174995	76454	6	12
MR266	SAMN18024931	28	40	317494	2832161	32.8	127819	73999	7	15
MR267	SAMN18024932	29	39	321836	2811773	32.72	150857	76452	7	12
MR268	SAMN18024933	38	38	406522	2852457	32.73	171342	77520	6	12
MR270	SAMN18024934	27	46	509512	2835744	32.65	133618	78821	7	14
MR277	SAMN18024935	30	20	498159	2667461	32.69	253207	114410	4	8
MR298	SAMN18024936	26	24	484924	2873694	32.71	204342	119547	5	9
MR3	SAMN18024937	37	17	659634	2717195	32.69	249701	191968	4	7
MR300	SAMN18024938	30	18	520548	2669471	32.83	408917	168770	3	6
MR320	SAMN18024939	28	31	465804	2708269	32.76	154322	127763	6	10
MR325	SAMN18024940	24	336	47595	2877024	32.73	14130	7656	64	134
MR33	SAMN18024941	28	22	723796	2743326	32.72	226732	169386	4	7
MR334	SAMN18024942	29	18	657028	2714520	32.7	315073	164057	3	6
MR349	SAMN18024943	30	22	590032	2750657	32.73	252207	193090	4	7
MR35	SAMN18024944	27	21	660586	2719601	32.69	243660	116268	4	8
MR36	SAMN18024945	28	75	251030	2773289	32.69	90675	41532	9	22
MR361	SAMN18024946	43	42	395630	2809903	32.74	150922	76411	6	12
MR396	SAMN18024947	31	37	435079	2852797	32.73	171342	99529	6	11
MR439	SAMN18024948	38	40	321836	2811708	32.72	150433	75060	7	13
MR440	SAMN18024949	28	11	870855	2786151	32.68	781415	206142	2	4

© 2021 Nurjadi D et al. *JAMA Network Open*.

MR446	SAMN18024950	31	23	547831	2731084	32.83	213203	137066	4	7
MR463	SAMN18024951	37	17	458659	2715632	32.73	318955	249958	4	6
MR470	SAMN18024952	30	31	280331	2803966	32.73	150966	112338	7	13
MR471	SAMN18024953	43	17	458659	2715386	32.72	318771	249958	4	6
MR472	SAMN18024954	54	18	458659	2716492	32.73	318771	249958	4	6
MR481	SAMN18024955	71	32	451815	2751082	32.7	159892	92428	5	11
MR492	SAMN18024956	36	29	343518	2759075	32.77	176713	71459	6	12
MR65	SAMN18024957	28	27	402727	2803299	32.72	198214	126843	5	10
MR76	SAMN18024958	34	17	793217	2718446	32.71	312646	173102	3	6

© 2021 Nurjadi D et al. *JAMA Network Open*.

eTable 2. *spa* Type Frequency

Transmission by WGS was defined as isolates with <20 SNPs, within the same *spa*-type group.

spa-type	freq. In study		global freq	% transmission by WGS
	n	%		
t002	4	3.15	6.57	0
t008	3	2.36	5.83	66.7
t084	8	6.3	1.74	44.4
t012	3	2.36	1.53	66.7
t021	5	3.94	1.07	80
t091	3	2.36	0.98	0
t223	2	1.57	0.61	100
t571	6	4.72	0.41	60
t330	4	3.15	0.15	100
t089	4	3.15	0.12	100
t254	4	3.15	0.07	80
t224	3	2.36	0.06	66.7
t803	2	1.57	0.06	100
t275	2	1.57	0.05	100
t153	3	2.36	0.04	100
t399	3	2.36	0.03	66.7
t589	2	1.57	0.03	100
t2235	3	2.36	0.02	100
t170	7	5.51	0.01	87.5
t2642	7	5.51	0.01	71.4
t1067	4	3.15	0.01	75
t1460	2	1.57	0.01	0
t2518	2	1.57	0.01	100
t3375	2	1.57	0.01	100
t6038	4	3.15	0.00	75
t2228	2	1.57	0.00	100
t489	2	1.57	0.00	100
t003	1	0.79	8.65	.
t127	1	0.79	2.62	.
t015	1	0.79	1.02	.
t304	1	0.79	0.57	.
t359	1	0.79	0.20	.
t645	1	0.79	0.20	.
t085	1	0.79	0.19	.

© 2021 Nurjadi D et al. *JAMA Network Open*.

t136	1	0.79	0.16	.
t040	1	0.79	0.13	.
t605	1	0.79	0.09	.
t156	1	0.79	0.08	.
t246	1	0.79	0.08	.
t095	1	0.79	0.05	.
t1255	1	0.79	0.05	.
t284	1	0.79	0.05	.
t505	1	0.79	0.04	.
t711	1	0.79	0.03	.
t163	1	0.79	0.02	.
t302	1	0.79	0.02	.
t748	1	0.79	0.01	.
t10097	1	0.79	0.00	.
t1644	1	0.79	0.00	.
t1758	1	0.79	0.00	.
t1869	1	0.79	0.00	.
t18877	1	0.79	0.00	.
t19202	1	0.79	0.00	.
t2013	1	0.79	0.00	.
t3272	1	0.79	0.00	.
t5386	1	0.79	0.00	.
t6889	1	0.79	0.00	.
t830	1	0.79	0.00	.

© 2021 Nurjadi D et al. *JAMA Network Open*.
